# Educating fathers to improve exclusive breastfeeding practices: a randomized controlled trial

**DOI:** 10.1186/s12913-022-07966-8

**Published:** 2022-04-26

**Authors:** Farideh Panahi, Farzaneh Rashidi Fakari, Soheila Nazarpour, Razieh Lotfi, Mitra Rahimizadeh, Maliheh Nasiri, Masoumeh Simbar

**Affiliations:** 1 Department of Midwifery and Reproductive Health, School of Nursing and Midwifery, Shahid Beheshti University of Medical Sciences, Tehran, Iran; 2 Department of Midwifery, School of Medicine, North Khorasan University of Medical Sciences-Bojnurd, Bojnurd, Iran; 3 Department of Midwifery, Chalous Branch, Islamic Azad University, Chalous, Iran; 4 Department of Midwifery, School of Nursing and Midwifery, Alborz University of Medical Sciences-Karaj, Karaj, Iran; 5 Department of Biostatistics, Social Determinant of Health Research Center, Alborz University of Medical Sciences-Karaj, Karaj, Iran; 6 Department of Basic Sciences, School of Nursing and Midwifery Shahid Beheshti University of Medical Sciences Tehran, Tehran, Iran; 7 Midwifery and Reproductive Health Research Center, Shahid Beheshti University of Medical Sciences, Vali-Asr Avenue, Cross of Vali-Asr and Hashemi Highway, Opposite to Rajaee Heart Hospital, Tehran, 1996835119 Iran

**Keywords:** Education, Exclusive breastfeeding

## Abstract

**Background:**

Fathers’ involvement is crucial for promoting breastfeeding. There are a few studies on the effectiveness of fathers’ educational programs to promote exclusive breastfeeding. This study aims to assess the effectiveness of a fathers’ educational program on their support for breastfeeding, mothers’ breastfeeding practice, and exclusive breastfeeding status.

**Methods:**

This was a randomized controlled trial on 76 fathers who were randomly assigned to two groups of intervention and control, in a selected health center in Iran, 2018.

The tools for data collection were: 1) a questionnaire for “Demographic and Maternal-Infant Information”; 2) a questionnaire to assess “Fathers’ support for Breastfeeding”, and 3) an observational checklist to assess “Mothers’ Breastfeeding Practice”; and 4) a questionnaire to assess “Exclusive Breastfeeding Status”. The questionnaires were filled up through an interview. The checklist was completed through observation by the researcher. The fathers (with the mothers) of the intervention group were educated using individual face-to-face education and counseling, in two sessions, with the duration of about 40 min and one-week interval, whereas, the fathers of the control group did not receive any education and only mothers were educated with the same instruction. The content of the education was: fathers’ education about “benefits of breast milk” and “the supporting ways for breastfeeding including the women encouragement”. Then, the scores of “father’s support for breastfeeding”, “mothers’ breastfeeding practice” and “exclusive breastfeeding status” were compared before and after 4 months of intervention in each group, and also between groups. Data were analyzed using SPPS-23, and t- and paired-tests, Chi-square, and Generalized-Estimating-Equations (GEE) tests.

**Results:**

The results showed two groups were not significantly different regarding the demographic and any other possible confounding variables before the intervention (*P* < 0.05). The before and after comparisons also demonstrated significant improvements in the two variables including “father’s support for breastfeeding”, and “mothers’ breastfeeding practice after 4 months, in the intervention group (Paired t-test: *P*<0.001 and *P*<0.0001, respectively) however, there was a significant decrease in “father’s support for breastfeeding” and no improvement in “mothers’ breastfeeding practice” after 4 months in the control group (Paired t-test: *P* < 0.001 and *P* = 0.07, respectively). Between groups comparison showed also significant higher scores for “father’s support for breastfeeding”, “mothers’ breastfeeding practice” and “exclusive breastfeeding status” in the intervention group comparing to the control group, after 4 months (T-test: *P* < 0.001 and *P* < 0.0001; Chi2: *P* < 0.001, respectively). The interaction effects of time and group were significant in the GEE test for the fathers’ support for breastfeeding (B-group = 31.93, B-time = 22.15, *p* < 0.001) and mothers’ breastfeeding practice (B-group = 26.32, B-time = 12.86, p < 0.0).

**Conclusion:**

The results showed that the father’s education improves mothers’ breastfeeding practice and increases the rate and continuity of exclusive breastfeeding.

**Trial registration:**

IRCT201508248801N10. “31/08/2016”.

**Supplementary Information:**

The online version contains supplementary material available at 10.1186/s12913-022-07966-8.

## Background

Breast milk provides all nutrients and necessary energy during the first months of life and breastfeeding is the best choice for optimal growth, development, and health of infants [1]. Exclusive breastfeeding is recommended to begin from the first hour after birth and continued to 6 months [2]. Breast milk guarantees the optimal physical and cognitive development of infants and protects them against infectious and chronic diseases [3]. Exclusive breastfeeding also decreases the rate of diarrhea, pneumonia, and infections [4].

A recent review study indicated that in 57 selected countries during 2010–2018, the global weighted prevalence was 45.7% for exclusive breastfeeding. Despite the importance of exclusive breastfeeding in low-income countries, only 45.1% percent of 6 months infants have exclusive breastfeeding [5]. In Iran, the estimated overall prevalence of exclusive breastfeeding was reported 27.7% in 2009 [6], 49.1% in 2016 [7], and 53% in 2019 [6] which are showing the improvement in the recent decade [8]. This improvement can be attributed to the developing baby-friendly hospitals and services and breastfeeding promotion programs in Iran [8]. A meta-analysis also showed an average duration of 4.1 months of exclusive breastfeeding in Iran [9]. However, according to the goals of the World Health Organization (WHO), the rate of exclusive breastfeeding should be increased to at least 60% by 2030 [10]. Besides, the American College of Obstetricians and Gynecologists recommends exclusive breastfeeding for the first 6 months of life to all women [11]. Also, based on Iranian Islamic culture and the Quran women are recommended to feed their babies for 24 months [7].

Although breastfeeding is an intuitive behavior, it is also an acquisitive behavior that can be improved by education and support [12, 13]. The majority of first-time breastfeeding mothers have inadequate knowledge and self-efficacy for breastfeeding [14, 15]. So, health care providers can improve mothers’ knowledge and behaviors through comprehensive education [16, 17]. Several demographic and psycho-social factors are shown to be related to the breastfeeding behavior of mothers such as maternal age, occupation, and the number of children as well as fathers’ support and encouragement [15, 18, 19].

The relationship between a father’s support for breastfeeding mother with improving rate of exclusive breastfeeding was shown in the previous studies [20, 21]. A qualitative study demonstrated that men are willing to help in maternal-fetal -neonatal care and breastfeeding, but they need knowledge and the appropriate perinatal education [22]. The association between fathers’ knowledge and attitude toward breastfeeding and the rate of exclusive breastfeeding highlights the importance of including fathers in the interventions for promoting breastfeeding [23]. In addition, it is found egalitarian attitudes towards parenthood were positively associated with both attitudes towards breastfeeding and levels of paternal involvement. It was similarly indicated fathers’ attitudes towards infant feeding were largely influenced by healthcare professionals [24]. Besides, the fathers need appropriate education and counseling services. A cross-sectional study showed that 95% of men agreed with paternal perinatal care education and preferred the face-to-face couples’ counseling method [25]. The studies showed that these services not only help them to adapt to paternal roles [26, 27] but also are effective in ensuring maternal-fetal and -neonatal health [28, 29]. Besides, recent studies have emphasized providing gender-based care and counseling services, especially based on the men’s needs in perinatal care and breastfeeding programs, to improve the quality of the services [30].

Although fathers’ support in improving breastfeeding is related to the success and continuity of breastfeeding [31, 32], inadequate information of men about the importance of breast milk is one of the most important barriers to their support [33]. It seems fathers’ education about the importance of breast milk for the infants’ growth and development may improve their support for breastfeeding mothers [34, 35].

Therefore, this study aims to assess the effectiveness of a fathers’ educational program on their support for breastfeeding, mothers’ breastfeeding practice, and exclusive breastfeeding status.

## Methods

### Design of the study

This was a randomized parallel-group controlled trial study on 76 fathers who attended a health center in Karaj-Iran.

### The participants

Inclusion criteria were assessed for 81 attendees to the health center using the criteria checklist. Five men were excluded as two of them had not the eligibility criteria, and 3 of them declined to participate. Seventy-six fathers were randomly assigned to the intervention (38 fathers) or control (38 fathers) groups. Samples were devoted to the control and intervention groups using the excel randomization option by the first author. They were recruited from attendees to a selected health center for receiving postpartum care services and had 3 to 5 days old neonates. The inclusion criteria were: men with a primiparous wife; with a healthy single neonate; lack of known medical condition and/or mental disorders (stated by the participant); and speaking in Persian. The exclusion criteria were: hospitalization of the neonate; posttraumatic stress disorder associated with the unexpected death of a loved one, or couple’s separation during the study period; taking medicine that prevents breastfeeding; using pacifier; and occurrence of unwanted pregnancy during the study period. The first author, Farideh Panahi (FP) assessed the eligibility criteria of the participants. Data collection was performed from June to December 2017.

### Sampling

The sample size was calculated by the rate of exclusive breastfeeding of 64 and 29% in the intervention and control groups in a similar study by Tavafian et al. [36], and considering 95% confidence interval and 80% power, using the following formula. The sample size was estimated at 30 and then considering 25% loss, 38 samples were devoted for each group.

$$n=\frac{{\left({Z}_{1-a/2}+{Z}_{1-\beta}\right)}^2\ast \left[{p}_1\left(1-{p}_1\right)+{p}_2\left(1-{p}_2\right)\right]}{{\left({p}_{1-}{p}_2\right)}^2}$$

### Blinding process

After devoting the participants to two groups of intervention and control by the researcher (FP), the questionnaires were coded by her. The data of the completed questionnaires were entered into SPSS and analyzed by a blind statistician (MR).

### Tools of the study

The tools for data collection were 4, including 1) a questionnaire to collect “Demographic and Maternal-Infant Information”; 2) a questionnaire to assess “Fathers’ support for Breastfeeding”, and 3) an observation checklist to assess “Mothers’ Breastfeeding Practice”; and 4) a questionnaire to assess “Exclusive Breastfeeding Status”. The questionnaires were filled up for the participants through an interview, by the first author (FP).

#### A questionnaire to collect “Demographic and Maternal-Infant Information”

This questionnaire consists of 14 questions to collect the participants’ demographic data and the maternal and infants’ information. The validity of the questionnaire was assessed and confirmed by 10 midwifery and reproductive health experts.

#### A questionnaire to assess “Fathers’ support for Breastfeeding”

The questionnaire consists of 12 items to measure fathers’ support for breastfeeding including items about mothers’ encouragement for breastfeeding; planning for nutrition and rest of mothers; the fathers’ involvement in housework and the infant-care. The items were assessed by a 5 point Likert scale from never to always (scoring 1 to five, respectively). The total scores were calculated and converted to percent, which indicated the level of Fathers’ support for breastfeeding. The validity of the questionnaire was assessed by 10 reproductive health experts. The validity was confirmed by S-CVI = 0.76 and S-CVR = 0.79, respectively. The reliability of the questionnaire was evaluated by assessing the internal consistency and the stability of the questionnaire. The internal consistency of the questionnaire was evaluated using Cronbach’s coefficient alpha. The coefficient of 0.93 for the entire questionnaire showed proper internal consistency. The stability of the questionnaire was evaluated using the test-retest stability assessment method. Fifteen men filled out the questionnaires within two-week intervals, and the Pearson correlation coefficient confirmed the stability of the questionnaire (r = 0.86; *p* < 0.05).

#### An observational checklist to assess “Mothers’ Breastfeeding Practice”

This checklist consists of 26 items to assess the breastfeeding practice of mothers; with “yes” or “no” responses (Scoring 1 and 0, respectively). The total score was calculated and converted to percent, which indicated the quality of mothers’ breastfeeding practice. The validity of the questionnaire was assessed by 10 reproductive health experts. The validity was confirmed by S-CVI = 0.78 and S-CVR = 0.83, respectively. The reliability of the checklist was confirmed by using the intra-rater stability assessment method. The breastfeeding practice of 15 mothers was rated by two researchers using the checklist, and then the average calculated ICC = 0.72 confirmed the stability of the checklist. Internal consistency assessment showed Cronbach’s α = 0.78 of the tool.

#### A questionnaire to assess “Exclusive Breastfeeding Status”

It contained 3 questions about exclusive breastfeeding.

### The intervention process

Before the intervention, both groups completed the “Demographic and Maternal-Infant Information” and “Fathers’ support for Breastfeeding” questionnaires*.* Then, fathers (with the mothers) of the intervention group were educated using individual face-to-face education and counseling, in two sessions, with a duration of about 40 min and one-week interval. The intervention was arranged in the second week and third week of their infant’s life. The content of the education was as below:

#### Intervention group

Fathers (with mothers) of the intervention group were educated about the composition of breast milk; the importance and the benefits of exclusive breastfeeding for maternal and neonatal health; the correct positions for breastfeeding, in the first session. In the second session, the fathers were educated about their critical role in promoting and continuing breastfeeding; the ways for mothers’ encouragement, or planning for regular exclusive breastfeeding; involving in housework and infant-care, to free adequate time for mothers’ rest. The education of fathers with mothers (couples) was conducted by the first author Mrs. Farideh Pnahi in the childbirth preparation classes.

#### Control group

Fathers in the control group did not receive any education and only mothers were educated with the same instruction, about the composition of breast milk; the importance and the benefits of exclusive breastfeeding for maternal and neonatal health, and the correct positions for breastfeeding.

Four months later, all fathers in both groups completed the “Fathers’ support for Breastfeeding” and “Mothers’ Breastfeeding Practice” questionnaires, again; as well as the” Exclusive Breastfeeding Status” questionnaire. No harm was reported from the fathers’ education as the intervention. Therefore, only mothers were educated in the control group by the first author Mrs. Farideh Panahi in the childbirth preparation classes of the centers. The process of the study and data collection was performed from June to December 2017.

### Data analysis

Data were analyzed using SPPS 23, and by statistical tests such as paired and t-test, Chi2. The Generalized Estimating Equation (GEE) test was used to evaluate the effects of time and groups on the outcomes of the study. The significance level was *p*-value lower than 0.05.

There were no changes to the method and outcome measures after trial commencement.

## Results

**Fig. 1:**
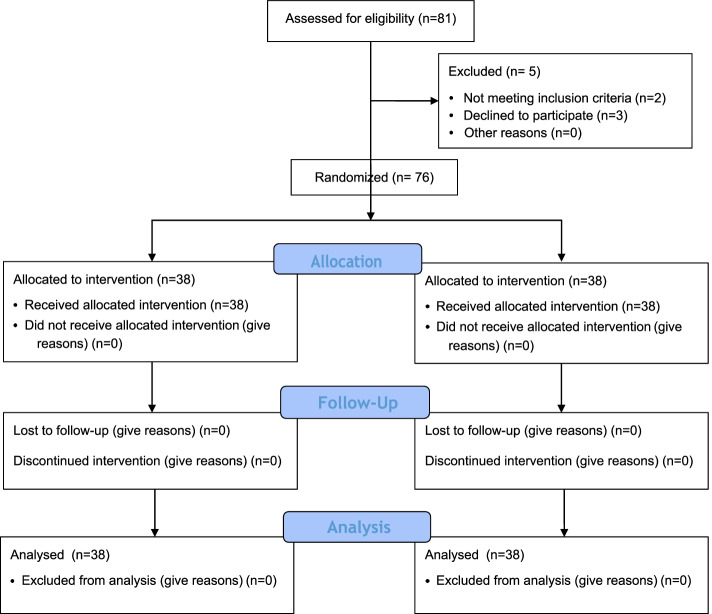
The Consort Flow Chart

Inclusion criteria were assessed for 81 attendees to the health center and 5 men were excluded; as two of them had not the eligibility criteria, and 3 of them declined to participate. Finally, 76 fathers accepted the condition and were recruited for the study. All 76 participants in the intervention and control groups completed the study and there was no drop–out within the groups (Fig. 1).

Demographic and maternal and neonate information of participants in two groups are shown in Table 1. The results showed two groups were not significantly different regarding the demographic and any other possible confounding variables (*P* < 0.05).

**Table 1:** The Comparison of demographic and maternal-infant health characteristics of two groups of the study

Variables		Control (*n* = 38)		Intervention (*n* = 38)		Test (*P* Value)
		Mean	SD	Mean	SD	
Age of father (Years)		29.36	8.01	29.31	5.92	0.49
Age of Mother (Years)		22.26	6.63	21.73	6.65	0.06
Weight of Neonate (gr)		3644	12.4	3684	5.4	0.6 **
High of Neonate (cm)		49.76	2.40	48.92	3.74	0.2 **
Apgar of 1 min		8.73	3.34	8.84	3.36	0.08 ***
Apgar after 5 min		9.92	0.59	9.97	0.23	0.1 ***
		No	%	No	%	
Fathers’ educational level	Primary School	12	31.61	12	31.61	0.6 ***
	High school	8	21.05	13	34.22	
	Academic	18	47.34	13	34.22	
Mothers’ educational level	Primary School	2	5.26	3	7.85	0.97 ***
	High school	17	44.71	16	42.1	
	Academic	19	50.05	19	50.05	0.13
Fathers’ Job	Unemployed	1	2.63	2	5.26	
	Farmer, Worker	5	13.1	6	15.76	
	Clerk	11	29.01	10	26.37	
	Shopper	21	55.26	20	52.61	
Maternal Job	House wife	36	94.8	38	100	0.06
	Employed	2	5.3	0	0	
Family’s monthly income	Adequate	7	18.42	9	23.68	0.1*
	Not adequate	31	81.58	29	76.32	
Initiation of breastfeeding after birth	Immediate	14	36.88	16	42.1	0.3*
	During the first hour	9	23.68	9	23.68	
	After one hour	15	39.47	13	34.21	
Neonate sex	Female	22	57.9	21	55.26	0.2*
	Male	16	42.1	17	44.71	

Tests: *Chi2; **Independent T test; ***Mann-Whitney u test

Results showed, no significant difference between groups respecting the “fathers’ support for breastfeeding” before intervention. But T-test analysis showed a significantly higher “Fathers’ support for Breastfeeding” among participants of the intervention group in comparison with the control group, after 4 months (*P* < 0.001) (Table 2).

**Table 2:** The comparison of “father’s support for breastfeeding” between groups, as well as before and after intervention

Time	Control (n = 38)		Intervention (n = 38)		Independent T test Between groups
	Mean	SD	Mean	SD	
Before intervention	76.45	17.75	70.65	17.61	***P*** **= 0.156; df = 74; t = 1.43**
Four months after intervention	60.87	9.99	92.8	7.02	***P*** **< 0.001; df = 74; t = −16.12**
Paired test (Intra-group)	***P*** **< 0.001; df = 37; t = 8.86**		***P*** **< 0.001; df = 37; t = 6.97**		

The intragroup comparison also demonstrated a significant decrease of fathers’ support for breastfeeding in the control group (Paired test; *P* < 0.001); while, a significant increase was shown among fathers of the intervention group, after 4 months (Paired test; *P* < 0.001) (Table 2).

Between groups comparison of “Mothers’ Breastfeeding Practice” showed no significant difference between groups before intervention (*P* = 0.7). However, there was a significantly higher score for Mothers’ Breastfeeding Practice in the intervention group compared to the control group, after the intervention, (T-test; *P* < 0.001) (Table 3).

**Table 3:** The comparison of “Mothers’ Breastfeeding Practice” between groups, as well as before and after intervention

The items^a^	Before				After			
	Control (*n* = 38)		Intervention (*n* = 38)		Control (*n* = 38)		Intervention (*n* = 38)	
	n	%	n	%	n	%	n	%
The mother is quite calm and comfortable	15	39.5	18	47.4	5	13.2	38	100
The infant’s face is in front of the mother’s breast	22	57.9	19	50	12	31.6	38	100
The infant’s head and body are in one direction	16	42.1	19	50	12	31.6	37	97.4
The infant’s chin is attached to the mother’s breast	20	52.6	17	44.7	16	42.1	38	100
The infant’s hips are in the mother’s arms	18	47.4	16	42.1	10	26.3	38	100
the infant sucks when she/he is hungry	18	47.4	22	57.9	24	63.2	38	100
The infant is properly sucking	21	55.3	23	60.5	22	57.9	38	100
There are signs of flowing milk	22	57.9	26	68.4	27	71.1	38	100
The mother embraces the infant with confidence	17	44.7	18	47.4	12	31.6	38	100
There is a face-to-face in maternal- infant relationship	16	42.1	19	50	14	36.8	38	100
The mother touches the infant while breastfeeding	16	42.1	17	44.7	12	31.6	36	94.7
Breasts are soft after breastfeeding	22	57.9	23	60.5	27	71.1	32	84.2
Nipples have adequate elasticity	22	57.9	20	52.6	19	50	38	100
The skin of the nipple is healthy.	18	47.4	23	60.5	32	84.2	38	100
Breasts look full when breastfeeding.	21	55.3	24	63.2	23	60.5	38	100
The infant takes both breasts without difficulty.	16	42.1	17	44.7	21	55.3	38	100
The infant is concentrated when breastfeeding.	22	57.9	24	63.2	21	55.3	38	100
The infant’s mouth is completely open.	19	50	21	55.3	25	65.8	38	100
The infant’s lower lips is turned outwards	22	57.9	22	57.9	30	78.9	38	100
The tongue surrounds the breast	23	60.5	23	60.5	23	60.5	38	100
Cheeks are Hollow and protruding	19	50	18	47.4	22	57.9	38	100
Most of the areola is seen above the infant’s mouth.	20	52.6	20	52.6	23	60.5	38	100
Sucking is slow and deep.	26	68.4	24	63.2	24	63.2	38	100
The sound of swallowing can be heard.	20	52.6	19	50	27	71.1	38	100
The infant releases the breasts by him/herself	21	55.3	22	57.9	18	47.4	38	100
The infant appears to be full after breastfeeding	21	55.3	21	55.3	18	47.4	38	100
Mean ± SD (Score 0–100)	54.15 ± 38.01		51.92 ± 33.42		52.53 ± 19.33		99.1 ± 1.66	
Paired test (Intra-Group Comparison)	Intra-Control group *P* = 0.7; T = 0.4; df = 37				Intra-intervention group *P* < 0.0001; t = −8.7; df = 37			
Independent T test (Between Groups Comparison)	Before (between control and intervention) *P* = 0.7; T = 0.27;df = 74				After (between control and intervention) *P* < 0.0001; T = -14.8;df = 74			

^a^These 26 items were used to assess breastfeeding practice of mothers; with “yes” or “no” responses, Scoring 1 and 0, respectively. The scoring as used to calculate mean score for mothers’ breastfeeding practice. The frequencies including numbers and percent show the frequencies of “yes” responses

Intra-groups comparison of “Mothers’ Breastfeeding Practice” also demonstrated no significant improvement in the control group after 4 months (*P* = 0.6), while there was a significant improvement in the intervention group, compared to before intervention (Paired test; *P* < 0.001) (Table 3).

Finding also showed a higher frequency of exclusive breastfeeding after 4 months in the intervention group compared to the control group after intervention (*P* < 0.001) (Table 4).

**Table 4:** The comparison of” Exclusive Breastfeeding Status” of two groups of study, as well as before and after intervention

Variables	Before				After			
	Control (***n*** = 38)		Intervention (***n*** = 38)		Control (***n*** = 38)		Intervention (***n*** = 38)	
	n	%	n	%	n	%	n	%
Breast Milk	10	26.3	17	44.7	8	21.1	32	84.2
Formula milk	3	7.9	1	2.6	3	7.9	5	13.2
Breast milk with formula	25	65.8	20	52.6	27	71.1	1	2.6
Chi^2^ test	***P*** **= 0.18; df = 2;PearsonChi2 = 3.37**				***P*** **< 0.001; df = 2; PearsonChi2 = 39.04**			

**Fig. 2:**
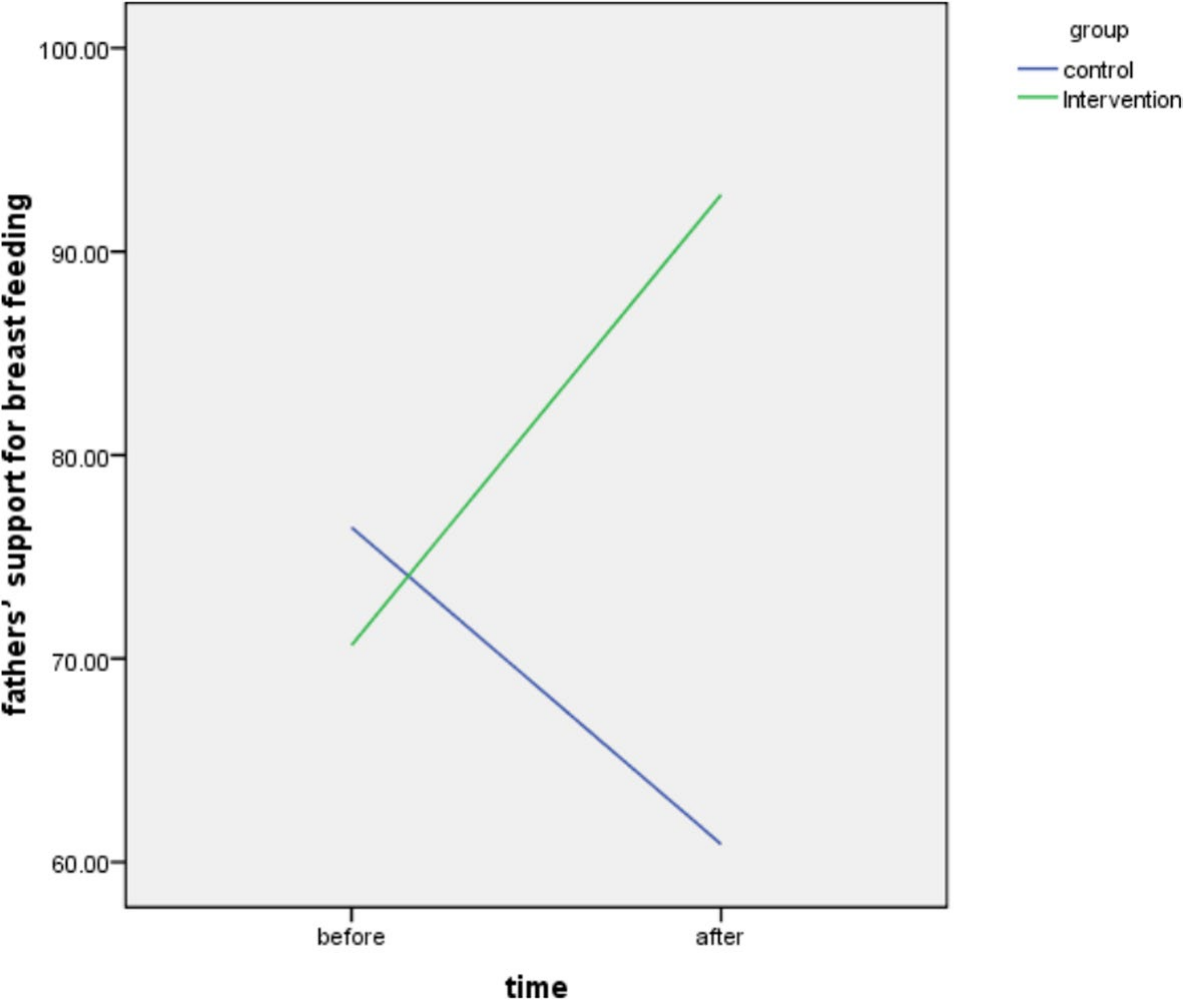
The comparison of the fathers’ supports for breastfeeding, before and after intervention in the control and the intervention groups

**Fig. 3:**
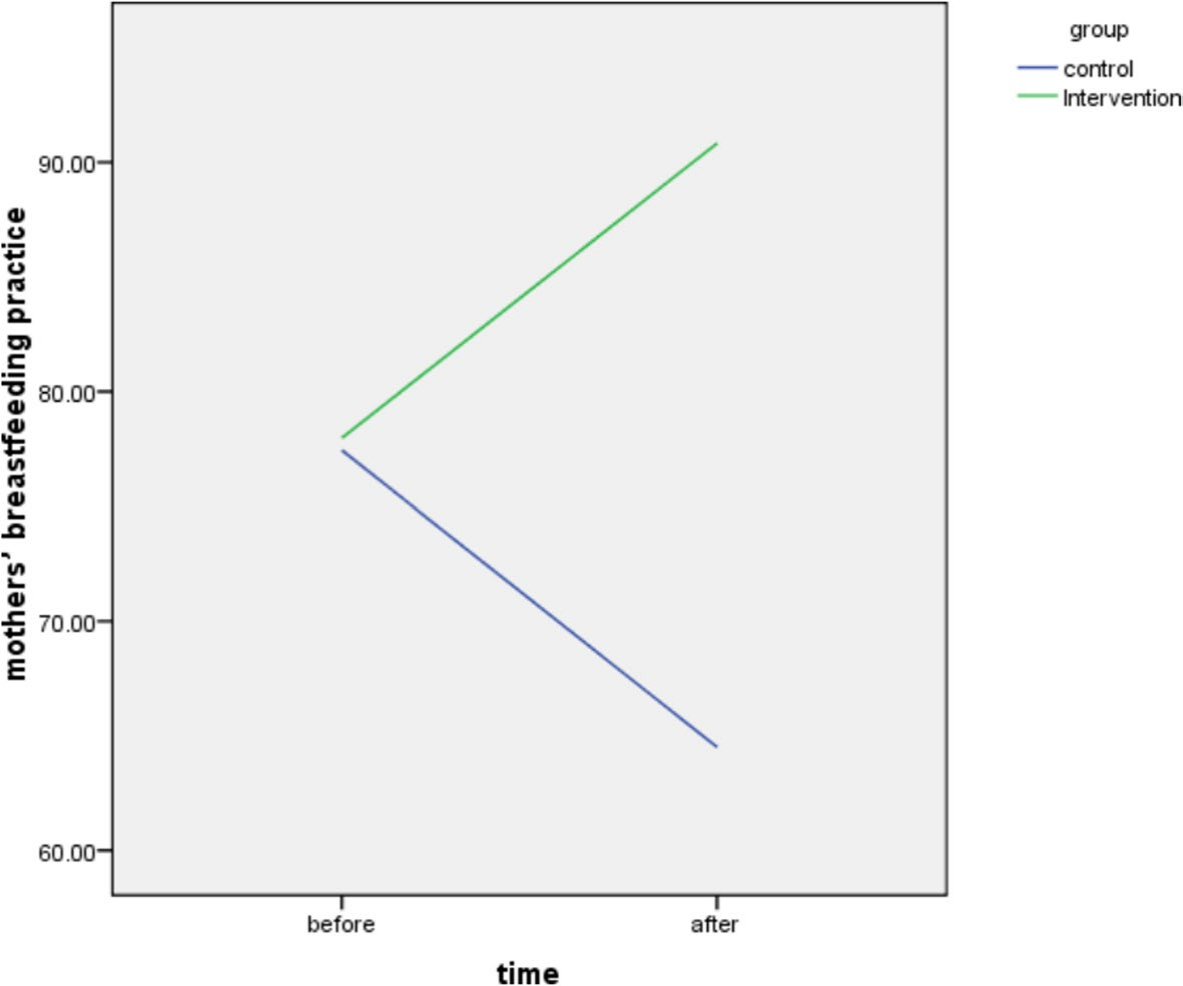
The comparison of mothers’ breastfeeding practice, before and after intervention in the control and the intervention groups

The results showed the interaction effects of time and group were significant in the GEE test for the fathers’ support for breastfeeding. The test results showed that the mean score of father’ support for the intervention group is increased 31.93 units more than the control group, and the mean score for fathers’ support after the intervention is increased by 22.15 units more than the before the intervention, and the interaction was significant (B-Intercept = 92.807; *P* < 0.001;df = 1) (Table 5). Besides, the interaction effects of time and group were also significant in the GEE test for the mothers’ breastfeeding practice as the test results showed that the mean score of mothers’ breastfeeding practice for the intervention group is 26.32 units more than the control group and the mean score of mothers’ breastfeeding practice after the intervention is 12.86 units more than before intervention (B-Intercept = 90.827; *P* < 0.01;df = 1) (Table 6). Figure 2 shows the comparison of the fathers’ support for breastfeeding, before and after the intervention in the control and the intervention groups. Figure 3 demonstrates the comparison of the mothers’ breastfeeding practice for breastfeeding, before and after intervention in the control and the intervention groups.

**Table 5:** Results of Generalized-Estimating-Equations (GEE) test to assess the interaction effects of time and group on the outcome of intervention on fathers’ support for breast feeding

Parameter Estimates							
Parameter	B	Std. Error	95% Wald Confidence Interval		Hypothesis Test		
			Lower	Upper	Wald Chi-Square	df	***P***
(Intercept)	92.81	1.12	90.61	95.01	6823.20	1	<0.001
Intervention group	31.93	1.95	28.10	35.76	266.902	1	<0.001
control group	reference	.	.	.	.	.	.
After	22.15	3.13	16.01	28.29	49.95	1	<0.001
Before	reference	.	.	.	.	.	.
group* time	−37.72	3.58	−44.74	−30.70	110.95	1	<0.001

Dependent Variable: Fathers’ support for breastfeeding

Model: (Intercept), group, time, group * time

**Table 6:** Results of Generalized-Estimating-Equations (GEE) test to assess the interaction effects of time and group on the outcome of the intervention on mothers’ breastfeeding practice

Parameter Estimates							
Parameter	B	SE	95% Wald Confidence Interval		Hypothesis Test		
			Lower	Upper	Wald Chi- Square	df	*P*
(Intercept)	90.83	1.23	88.42	93.23	5479.45	1	<0.001
Intervention group	**26.32**	2.24	21.93	30.71	138.02	1	<0.001
control group	reference	.	.	.	.	.	.
After	**12.86**	2.50	7.97	17.75	26.55	1	<0.001
Before	reference	.	.	.	.	.	.
group* time	**−25.79**	3.49	−32.63	−18.95	54.54	1	<0.001

Dependent Variable: Mothers’ breastfeeding practice

Model: (Intercept), group, time, group * time

## Discussion

This study showed the effectiveness of an educational program for fathers to support breastfeeding practice and exclusive breastfeeding continuity. Many studies are showing the effectiveness of educational interventions for promoting breastfeeding behaviors. However, a few studies have examined the effects of fathers’ education on breastfeeding practice and exclusive breastfeeding behaviors in Iran. This Iranian study showed and confirmed the results of other studies that fathers’ education can improve their involvement and support that lead to improving breastfeeding behaviors and exclusive breastfeeding promotion [37].

The results demonstrated that the fathers’ education to support breastfeeding improves their engagement in encouragement and planning a successful breastfeeding practice. A study in Iran showed that the majority of fathers are willing to participate in reproductive health and perinatal care and promote breastfeeding, however, they do not know; how to support breastfeeding [25]. A qualitative study showed the priority for improving fathers’ participation in perinatal care is their education about the ways for psychological support in the perinatal period. The participants stated that “men can show their support by some behaviors such as providing the necessary nutrients for the wife, facilitating the condition for the mother’s rest, and providing the necessary care and nutritional advice during perianal and breastfeeding period [22]. Further needs assessment seems to be required to determine special educational needs for improving fathers’ supportive behaviors for improving mothers’ breastfeeding practice.

In the present study, the participants learned to support mothers, for example through encouragement and planning for appropriate maternal nutrition and rest. The intervention was also concentrated on fathers’ contributions to housework and childcare, for decreasing daily tasks of breastfeeding mothers, and so manage time for mother’s adequate rest and thus preparing them for the night feeding. Subjects of the study were also taught about planning for regular and continuous exclusive breastfeeding. Targeting men in the breastfeeding educational programs was documented to be effective in the promotion of exclusive breastfeeding [38] A review study showed a variety of high- and low-intensity men’s involvement strategies that increase the prevalence of exclusive breastfeeding in different countries. High-intensity strategies engaged men directly during home or health visits. Low-intensity strategies included large community groups, radio messages, and other behavior change materials directed towards men [39]. A study also concluded that the most effective breastfeeding support is delivered using a sensitive, coordinated teamwork approach that is responsive to the mother’s needs [38].

The results demonstrated that the fathers’ education to support breastfeeding improves mothers’ breastfeeding practice. It is documented that social support especially, husband support increases self-efficacy in performing health behavior including breastfeeding behavior [15]. Also, men’s education about their roles and involvement in breastfeeding promotion increases mothers’ satisfaction and practice [40]. Previous studies demonstrated that fathers’ educational intervention about breastfeeding improves fathers’ knowledge and attitude towards breast milk and breastfeeding [41] and then they plan and encourage their nursing wives for breastfeeding [42].

The results also demonstrated a higher frequency for exclusive breastfeeding and a lower frequency of using formula after 4 months in the intervention group compared to the control group. Otherwise, the study showed longer continuity in exclusive breastfeeding after the education of fathers to support breastfeeding. It seems the education leads to promote exclusive breastfeeding behavior. This is consistent with the results of other studies in brazil and Turkey that showed fathers’ education about breastfeeding increases the duration of exclusive breastfeeding [41, 43]. There are many problems and barriers for continuity of exclusive breastfeeding such as misbeliefs about “inadequacy of milk for infant”, “deforming breast shape”, “difficulty for breastfeeding in social settings”, “difficulty in breastfeeding for employed mothers” [16]. The present study attempted to correct their beliefs and also recommend strategies to overcome the barriers. Also, education about the importance and benefits of breast milk for maternal and infant health helps couples for making appropriate decisions [44].

The findings of the present study showed couples’ education about breastfeeding can improve exclusive breastfeeding. This effect can be attributed to the importance of fathers’ supportive role in the primary stages of the postnatal period [21, 32]. It should be also noted that male involvement is not only helping to promote maternal-neonatal health but also is the main stage for paternal adaptation [26] and improves men’s health as well [25]. Therefore, paternal health and counseling services should be integrated into maternal-infant health services, and providing gender-based health services are strongly recommended for any society [30].

A limitation of the study was that in the intervention group, fathers were educated with the mothers about breastfeeding, which may affect mothers themselves learned from the sessions and implemented their new learning. However, as we explained in the method section; “Fathers in the control group did not receive any education about breastfeeding and only mothers were educated with same instruction”. Besides, the intragroup comparison by paired test showed no significant difference in the mothers’ practice after 4 months in the control group. Therefore, it could be concluded that fathers’ education with mothers about breastfeeding was effective on mothers’ practice. It is suggested that the replacement of maternal-paternal-neonatal services instead of the classic providing of the maternal-neonatal services may lead to better outcomes in breastfeeding promotion [23].

This study similar to other experimental studies had difficulty preventing the drop of subjects during the follow-up period. The researchers overcame this limitation using continuous contact with the participants.

## Conclusion

This study documented the effectiveness of the father’s education about the benefits of breast milk and the supporting ways for breastfeeding on improving their support, mothers’ breastfeeding practice, and increasing the rate and continuity of exclusive breastfeeding. So, fathers’ education about breastfeeding is recommended to be integrated into maternal health services, to promote male involvement in breastfeeding promotion.

Finally, the findings of this study consistent with previous studies [45] indicated that men’s participation in reproductive health and their acceptance of equal parental responsibilities in the family leads to the promotion of reproductive health, including the improving of exclusive breastfeeding status. Promoting men’s participation in reproductive health requires education not only at the community level but also at the level of the health system to promote family health including maternal-fetal and infant health.

## Supplementary Information

**Additional file 1.** Fathers’ Support for breastfeeding’ Questionnaire.

**Additional file 2.** The Observational Checklist to assess Mothers’ Breastfeeding Practice.

## Data Availability

All relevant raw data will be freely available to any scientist wishing to use them for non-commercial purposes, without breaching participant confidentiality. The study data are available by contacting the corresponding author of this paper.

## Abbreviations

GEE: Generalized-Estimating-Equations
WHO: World Health Organization
S-CVI: Scale Content Validity Index
S-CVR: Scale Content validity ratio
ICC: Intraclass correlation coefficient
