# Supplementary material for: Educating fathers to improve exclusive breastfeeding practices: a randomized controlled trial
Source: BMC Health Serv Res. 2022 Apr 26;22:554. doi: 10.1186/s12913-022-07966-8 (PMC9040207; doi:10.1186/s12913-022-07966-8)
Supplement: Supplementary file 1 — Additional file 1. Fathers’ Support for breastfeeding’ Questionnaire. [file 12913_2022_7966_MOESM1_ESM.docx]

| No | Item | Never | Seldom | Sometimes | Often | Always |
| --- | --- | --- | --- | --- | --- | --- |
| 1 | I remind my wife about eating nutrients like milk, liquids, fruits and vegetables, meat and eggs. |  |  |  |  |  |
| 2 | I remind my wife about eating meals and using different food groups. |  |  |  |  |  |
| 3 | I facilitate the conditions for my wife to rest during the night. |  |  |  |  |  |
| 4 | During the day and night, I remind my wife of the necessary tips on how to breastfeed properly. |  |  |  |  |  |
| 5 | I help my wife with household chores to facilitate her breastfeeding. |  |  |  |  |  |
| 6 | I help take care of my baby while my wife is resting. |  |  |  |  |  |
| 7 | In public places I seek for proper place for breastfeeding. |  |  |  |  |  |
| 8 | I remind my wife of the benefits of breast milk. |  |  |  |  |  |
| 9 | I remind my wife of the disadvantages of using formula |  |  |  |  |  |
| 10 | I remind my wife of the correct position to hold the baby |  |  |  |  |  |
| 11 | I remind my wife how to breastfeed properly. |  |  |  |  |  |
| 12 | I wake my wife up during the night to breastfeed |  |  |  |  |  |

**Fathers’ Support for breastfeeding’ Questionnaire**
