# Supplementary material for: Educating fathers to improve exclusive breastfeeding practices: a randomized controlled trial
Source: BMC Health Serv Res. 2022 Apr 26;22:554. doi: 10.1186/s12913-022-07966-8 (PMC9040207; doi:10.1186/s12913-022-07966-8)
Supplement: Supplementary file 2 — Additional file 2. The Observational Checklist to assess Mothers’ Breastfeeding Practice. [file 12913_2022_7966_MOESM2_ESM.docx]

| No. |  | Yes | No |
| --- | --- | --- | --- |
| 1 | The mother is quite calm and comfortable |  |  |
| 2 | The infant's face is in front of the mother's breast |  |  |
| 3 | The infant's head and body are in one direction |  |  |
| 4 | The infant's chin is attached to the mother's breast |  |  |
| 5 | The infant's hips are in the mother's arms |  |  |
| 6 | the infant sucks when she/he is hungry |  |  |
| 7 | The infant is properly sucking |  |  |
| 8 | There are signs of flowing milk |  |  |
| 9 | The mother embraces the infant with confidence |  |  |
| 10 | There is a face-to-face in maternal- infant relationship |  |  |
| 11 | The mother touches the infant while breastfeeding |  |  |
| 12 | Breasts are soft after breastfeeding |  |  |
| 13 | Nipples have adequate elasticity |  |  |
| 14 | The skin of the nipple is healthy. |  |  |
| 15 | Breasts look full when breastfeeding. |  |  |
| 16 | The infant takes both breasts without difficulty. |  |  |
| 17 | The infant is concentrated when breastfeeding. |  |  |
| 18 | The infant's mouth is completely open. |  |  |
| 19 | The infant's lower lips is turned outwards |  |  |
| 20 | The tongue surrounds the breast |  |  |
| 21 | Cheeks are Hollow and protruding |  |  |
| 22 | Most of the areola is seen above the infant's mouth. |  |  |
| 23 | Sucking is slow and deep. |  |  |
| 24 | The sound of swallowing can be heard. |  |  |
| 25 | The infant releases the breasts by him/herself |  |  |
| 26 | The infant appears to be full after breastfeeding |  |  |

**The Observational Checklist to assess Mothers’ Breastfeeding Practice**
